# Robotic-Assisted Surgery Adoption: A Mixed-Methods Evaluation of Digital Education Strategies and Surgical Training Needs

**DOI:** 10.7759/cureus.99505

**Published:** 2025-12-17

**Authors:** Nagesh Ramanath, Harsh H, Manish Kumar, Sudharshan Sampath, Eleyaraja Jagadeesan

**Affiliations:** 1 Business Administration, Indian Institute of Management Kozhikode, Kozhikode, IND; 2 Anesthesia and Intensive Care, Kauvery Hospital - Bangalore, Bengaluru, IND

**Keywords:** digital education, health literacy, mixed-methods research, patient perceptions, robotic-assisted surgery, surgical training

## Abstract

Background: The widespread adoption of robotic-assisted surgery (RAS) faces significant barriers, including a substantial public knowledge gap and a lack of formalized training pathways for surgeons.

Objective: The primary objectives were to evaluate the public's awareness and understanding of RAS, identify the prevalent patient misconceptions and surgical training deficits through expert interviews, and, based on these findings, develop and pilot-test a multi-tiered digital education platform.

Methods: A sequential mixed-methods design was employed. The quantitative phase involved a national survey of 507 participants in India to assess RAS knowledge and demographic correlations. The qualitative phase consisted of in-depth, semi-structured interviews with nine robotic surgeons to identify common patient misconceptions and training needs. The insights from both phases were triangulated to inform the design of a three-tiered digital education platform, which was subsequently pilot-tested with a small cohort of participants to evaluate its efficacy.

Results: The survey revealed a significant discrepancy between public awareness (n = 452, 89.2%) and functional understanding (n = 88, 17.3%) of RAS. Expert interviews confirmed this gap, with surgeons (n = 8, 89% of respondents) reporting the belief in autonomous robotic operation as the most common misconception. The qualitative data also highlighted a critical deficit in surgical training, with early-career surgeons averaging only 12.4 console cases. The pilot test of the developed digital platform demonstrated a 43% overall increase in patient understanding, with comprehension of the surgeon’s control mechanisms increasing by 100% (from n = 21 (41%) to n = 41 (82%)).

Conclusion: The findings confirm that a significant gap exists between RAS adoption and public understanding, a problem exacerbated by deficiencies in surgical training.

## Introduction

Robotic-assisted surgery (RAS) has fundamentally transformed modern surgical practice through its integration of advanced technologies and minimally invasive techniques. The clinical adoption of RAS has demonstrated significant improvements in surgical precision, particularly in complex oncologic procedures where studies have shown superior yields compared to traditional approaches [1]. The technological foundation of RAS platforms, including articulating instruments with high-definition three-dimensional visualization, provides surgeons with enhanced operative capabilities that were previously unattainable [2]. These advancements have been particularly impactful in specialties requiring meticulous dissection and reconstruction, where the improved visualization and instrument control have translated into measurable clinical benefits [3].

The comparative effectiveness of RAS has been extensively studied across multiple surgical disciplines. In urologic oncology, randomized controlled trials have demonstrated that robotic-assisted radical prostatectomy achieves significantly lower rates of positive surgical margins when compared to laparoscopic techniques [4]. General surgery applications reveal a more nuanced picture, with robotic cholecystectomy showing equivalent safety profiles to laparoscopic approaches despite longer operative times [5]. The learning curve associated with RAS varies substantially by procedure complexity, with multi-institutional studies documenting proficiency depending on the surgical complexity. These findings underscore the importance of procedure-specific training and the need for standardized metrics to assess surgical proficiency in robotic techniques [6].

The widespread adoption of RAS faces several significant barriers that extend beyond technological considerations. Surgical training represents a particularly critical challenge, as current residency programs struggle to provide adequate robotic experience [7]. Patient perceptions and understanding of RAS present another important barrier, with surveys indicating that less than half of patients correctly understand the surgeon's role in controlling robotic systems. This knowledge gap varies significantly by education level, highlighting the need for targeted patient education initiatives [8].

Recent innovations in RAS technology are addressing longstanding limitations while creating new possibilities for surgical care. Artificial intelligence integration has emerged as a particularly promising area, with systems now capable of providing real-time anatomical recognition and surgical navigation [9]. The development of improved haptic feedback mechanisms is helping to overcome the traditional limitation of reduced tactile sensation in robotic systems [10]. Additionally, the miniaturization of robotic platforms is enabling less invasive single-port access approaches that may further reduce surgical trauma [11]. These technological developments are currently undergoing rigorous clinical evaluation, with preliminary results suggesting significant potential to reduce operative variability and improve consistency in surgical outcomes.

The current evidence base supports RAS as an important advancement in surgical care, though its optimal implementation requires addressing several key challenges. Standardized training protocols must be developed and validated through multicentre trials to ensure consistent surgical proficiency [12]. Additionally, comprehensive cost-effectiveness analyses from healthcare system perspectives will be essential for guiding appropriate resource allocation. As robotic platforms continue to evolve, their integration with emerging technologies like artificial intelligence promises to further enhance surgical capabilities, though this potential must be carefully balanced against the need for rigorous clinical validation and equitable access. This study focuses specifically on elucidating and addressing the informational and educational prerequisites for patient engagement with RAS, acknowledging that systemic factors like cost, infrastructure, and policy represent separate, though interrelated, challenges to widespread implementation.

The present study aims to (1) assess public awareness and understanding of RAS through a stratified national survey; (2) evaluate surgical professionals' perspectives on implementation challenges; (3) analyze the effectiveness of current training paradigms; and (4) develop an evidence-based framework for digital patient education platforms. Our objectives include identifying knowledge gaps among diverse demographic groups, establishing procedure-specific learning curves, and proposing standardized metrics for surgical proficiency assessment. The ultimate goal is to create a comprehensive digital solution that bridges the gap between clinical evidence and patient understanding while addressing barriers to equitable access.

## Materials and methods

Study design

The present study employed a sequential explanatory mixed-methodology to comprehensively analyze and evaluate the potential for a digital platform addressing RAS education. The methodology was structured across four distinct phases, combining quantitative survey data with qualitative expert interviews to ensure robust and multi-dimensional findings. The approach was designed to capture both broad patterns in public awareness and nuanced insights from surgical professionals, creating a holistic understanding of the current RAS education landscape and digital solution requirements.

Phase 1: quantitative public survey

Survey Development and Validation

A 35-item survey instrument was developed to assess public awareness, knowledge, and perceptions of RAS. The instrument was structured into four domains: (1) Demographic Information, (2) General Awareness and Information Sources (5 items), (3) Knowledge of RAS Principles and Misconceptions (15 items; e.g., “The robotic system operates autonomously without the surgeon’s control”), and (4) Educational Preferences and Decision-Making Factors (10 items). Content validity was established through an expert panel review with three robotic surgery specialists, yielding a Scale-Level Content Validity Index (S-CVI) of 0.90. The instrument was then pilot-tested with a separate sample of 50 participants. Analysis of the pilot data confirmed the internal consistency of the multi-item knowledge domain, with a Cronbach’s α of 0.78. The final survey instrument is provided in Appendix A.

Sampling and Data Collection

This cross-sectional study employed a stratified convenience sampling approach to recruit a demographically diverse sample of the Indian population. Recruitment strata were defined a priori based on key variables: age group (18-22, 23-42, 43+ years), gender, and geographic location (metropolitan, tier-2/3 cities, semi-urban, rural). Quotas were set for each stratum proportional to recent national census data to guide recruitment. Primary data collection occurred over four weeks via online distribution through professional networks and health information portals. To mitigate the bias of limited internet access in rural regions, recruitment was supplemented through collaborations with community health centers in target areas, which facilitated survey access via provided tablets. Table 1 summarizes the final participant distribution.

**Table 1:** Participant Demographics, RAS Awareness, Understanding, and Educational Interest (N = 507) Percentages for Awareness, Understanding, and Interest are calculated within each demographic category (e.g., 49/55 = 89.1%). The "n (%)" column shows the composition of the sample. RAS: robotic-assisted surgery

Demographic Variable	Category	n (%)	Aware of RAS, n (% Within Category)	Accurate Understanding, n (% Within Category)	Interest in Digital Education, n (% Within Category)
Total Sample	-	507 (100)	452 (89.2)	88 (17.3)	400 (78.9)
Age Group	18-22 years	55 (10.8)	49 (89.1)	5 (9.1)	50 (90.9)
	23-42 years	334 (65.9)	298 (89.2)	72 (21.6)	277 (82.9)
	43+ years	118 (23.3)	105 (89.0)	11 (9.3)	73 (61.9)
Geographic Residence	Urban (Metro/Tier-2/3)	441 (87.0)	421 (95.5)	87 (19.7)	354 (80.3)
	Rural/Semi-urban	66 (13.0)	31 (47.0)	1 (1.5)	46 (69.7)
Education Level	College Graduate	304 (60.0)	289 (95.1)	88 (28.9)	268 (88.2)
	Non-graduate	203 (40.0)	163 (80.3)	0 (0.0)	132 (65.0)

Inclusion criteria required participants to be adults (≥18 years), residents of India, and able to provide informed consent. Medical professionals were excluded to avoid bias from prior clinical knowledge. All participants provided digital informed consent before proceeding. Data quality was maintained through built-in attention-check questions and logical validation rules within the survey platform.

Platform Pilot Test Methodology

Following the development of the three-tiered digital education platform, a separate pilot evaluation was conducted to assess its efficacy. A new convenience sample of 50 participants was recruited based on the following criteria: age ≥18, non-medical professional, and self-reported limited prior knowledge of RAS (screening score of ≤4 correct on a 10-item baseline knowledge test). This pilot sample did not overlap with the main survey cohort.

A pre-test/post-test design was implemented. Participants first completed a 10-item knowledge assessment covering key concepts addressed by the platform (e.g., surgeon’s role, advantages, safety). They then engaged with the core modules of the digital platform for a minimum of 20 minutes. Immediately following this engagement, participants completed the same 10-item knowledge assessment as a post-test. The primary outcome was the percentage change in correct responses. The knowledge assessment instrument used for this pilot is provided in Appendix B.

Phase 2: qualitative expert interviews

The qualitative component involved in-depth, semi-structured interviews with nine robotic surgeons selected through purposive sampling to ensure representation across surgical specialties (urology: n = 2, orthopaedics: n = 3, gynaecology: n = 2, general surgery: n = 2) and clinical experience levels (early career: n = 5, mid-career: n = 3, senior: n = 1). Interviews, lasting 45-60 minutes each, were conducted via secure video conference using a guide developed from the survey findings and literature. The guide covered four key domains: (1) clinical experiences with patient misconceptions, (2) recommendations for digital education features, (3) implementation challenges for RAS, and (4) ethical considerations. All interviews were audio-recorded with consent and professionally transcribed verbatim.

Phase 3: qualitative data analysis

Interview transcripts were analyzed using inductive thematic analysis. Analysis was facilitated by the qualitative data management software NVivo (version 12; QSR International, Melbourne, Australia). The coding process involved two primary researchers:

Familiarization and Open Coding

Both researchers independently read all transcripts and applied initial open codes to capture key concepts.

Development of a Coding Framework

Through interactive discussion, the researchers consolidated open codes into a structured codebook with definitions and examples.

Systematic Coding and Reliability

The two researchers then independently recoded three transcripts (33% of the data) using the agreed-upon codebook. Inter-coder reliability was assessed using Cohen's Kappa, yielding a score of 0.82, indicating strong agreement. Discrepancies were resolved through consensus, and the final codebook was applied to the remaining transcripts.

Theme Development and Validation

Coded data were analyzed for patterns, leading to the generation of initial themes. Thematic saturation was determined when analysis of the final two interviews yielded no new substantive codes or thematic insights. To enhance credibility, member checking was performed by sharing a summary of the preliminary themes with four interview participants via email; their confirmatory feedback was incorporated into the final thematic structure.

Chi-square tests were used for exploratory comparisons of awareness and understanding across demographic categories without adjustment for multiple comparisons, as the primary aim was descriptive. Primary analysis of factors associated with an accurate understanding was conducted using binary logistic regression (Table 2).

**Table 2:** Predictors of Accurate Understanding of RAS (Binary Logistic Regression, N = 507) The dependent variable was an accurate functional understanding of RAS (1 = Yes, 0 = No). The model demonstrates that higher education and urban residence are significant independent predictors of accurate understanding. RAS: robotic-assisted surgery

Predictor	Category (Reference)	Odds Ratio (OR)	95% Confidence Interval	P-value
Education Level	College Graduate (vs. Non-graduate)	2.31	1.65-3.23	<0.001
Residence	Urban (vs. Rural/Semi-urban)	1.92	1.24-2.98	0.003
Age Group	23-42 years (vs. 18-22 years)	1.45	0.89-2.36	0.134
	43+ years (vs. 18-22 years)	1.21	0.72-2.03	0.475
Model Summary		χ^2^ (4) = 38.2, p < 0.001		

Phase 4: digital platform development and pilot testing

**Figure 1:**
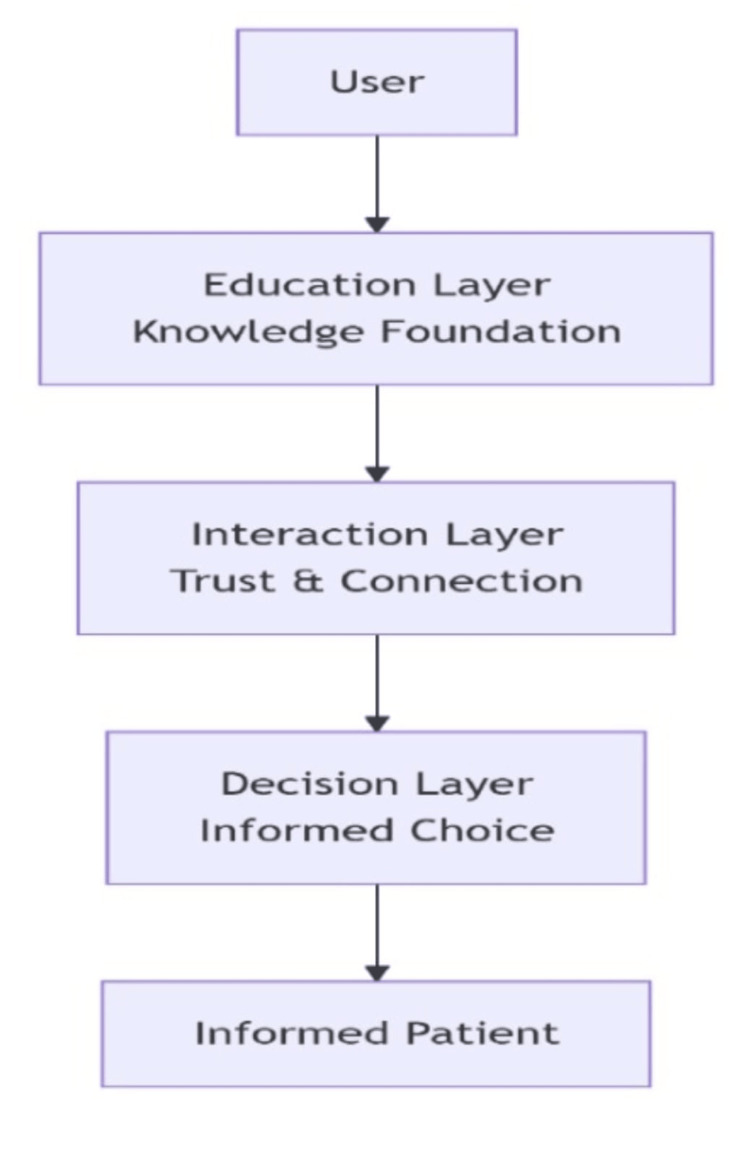
Schematic Representation of the Three-Tiered Digital Education Platform

The insights derived from the quantitative survey and qualitative interviews were systematically triangulated to inform the design and development of a three-tiered digital education platform. The architecture was established through an iterative design process, culminating in a high-fidelity interactive prototype constructed for user interface (UI) design and logic flow, with the overall structure illustrated in Figure 1.

The Education Layer (Foundational Knowledge) was designed to directly counter identified knowledge gaps and misconceptions. Content for this layer was developed by a multidisciplinary team comprising robotic surgeons, medical illustrators, and health communication specialists. Its core features include short-form explainer videos (under three minutes) to debunk myths such as robotic autonomy, interactive WebGL-based 3D anatomy visualizers to demonstrate procedural applications, and shareable myth-versus-fact cards. The Interaction Layer (Trust and Connection) was engineered to facilitate direct engagement between patients and the healthcare system, featuring a searchable directory of verified surgeon profiles with credentials and patient testimonials, an integrated teleconsultation scheduler, and a secure portal for submitting questions. Finally, the Decision Layer (Informed Choice) provides tools to support personalized decision-making, incorporating a dynamic cost comparator for procedure options, a dashboard visualizing aggregated clinical outcome data, and a personalized information packet generator that compiles user-selected content into a PDF report for clinician discussion (see Appendix C).

The platform's efficacy was rigorously assessed through a two-stage validation process. First, expert content validation was conducted: five independent robotic surgeons and two patient education specialists reviewed the content and design, rating accuracy, clarity, and usefulness on a 5-point Likert scale. The S-CVI was calculated as the proportion of items rated 4 or 5 by all experts, yielding scores of 0.89 for the Education Layer, 0.92 for the Interaction Layer, and 0.93 for the Decision Layer. Second, pilot testing was conducted with a separate convenience sample of 50 participants (adults, non-medical, with limited prior RAS knowledge). Utilizing a pre-test/post-test design with the 10-item knowledge assessment from Appendix B, participants engaged with the prototype for 20 minutes. Usability and satisfaction were measured via a post-engagement survey using items adapted from the System Usability Scale (SUS), which yielded an average score of 82.4, indicative of "good" to "excellent" usability. It is important to clarify that the Cronbach’s α values reported in the initial submission pertained specifically to the internal consistency of the satisfaction survey items related to the perceived usefulness of each platform layer; these metrics are now appropriately positioned within the pilot testing results.

Ethical approval

The study adhered to stringent ethical standards throughout all phases. Formal approval was obtained from the Kauvery Institutional Ethics Committee (Reg. No. EC/NEW/INST/2023/3361) on 14 June 2023, prior to the commencement of the study. Written informed consent was secured from all participants. Robust data protection measures were implemented, including secure servers with encryption, compliance with the Health Insurance Portability and Accountability Act (HIPAA) and the General Data Protection Regulation (GDPR), and complete anonymization of all participant data. To address potential biases, researchers maintained reflexivity journals documenting personal perspectives and potential influences, conducted regular peer debriefing sessions, and established comprehensive audit trails for all qualitative analysis procedures. These measures ensured the research maintained the highest ethical standards while protecting participant rights and data confidentiality.

Validity and reliability measures

Multiple measures were implemented to ensure the validity and reliability of findings. For the quantitative component, these included extensive pilot testing of the survey instrument, test-retest reliability assessments, and sampling adequacy tests. Qualitative validity was ensured through prolonged engagement over a six-month study period, negative case analysis to account for divergent perspectives, and thick description of findings to provide context-rich interpretations. These complementary approaches provided multiple lenses through which to verify the consistency and accuracy of the collected data, strengthening confidence in the study's conclusions.

## Results

Demographic breakdown of RAS understanding

Demographic breakdown of RAS understanding revealed significant variations across different groups, as demonstrated in Table 1. A chi-square test of independence revealed a significant association between geographic residence and awareness level, χ^2^ (3, N = 507) = 85.34, p < 0.001, with a medium effect size. While nominal awareness about RAS was generally high, it was highest among urban residents (93%) and college graduates (95%), compared to rural residents (68%). However, accurate understanding remained low across all groups, with notable disparities evident between urban and rural residents and across education levels. Interest in digital education was consistently high, particularly among younger and college-educated participants.

RAS awareness and understanding: a nationwide survey analysis

As summarized in Table 1, a comprehensive survey of 507 participants across India revealed a significant gap between nominal awareness of RAS (n = 452, 89.2%) and functional understanding of its principles (n = 88, 17.3%). Predictors of accurate understanding were analyzed using binary logistic regression. The model, containing age group, urban residence, and education level, was statistically significant (χ^2 ^(3) = 38.2, p < 0.001). Education level (odds ratio (OR) = 2.31, 95% confidence interval (CI) (1.65, 3.23), p < 0.001) and urban residence (OR = 1.92, 95% CI (1.24, 2.98), p = 0.003) were significant predictors, while age group was not (p = 0.15).

Demographic variations in educational preferences

Detailed analysis of learning preferences revealed significant differences across population subgroups. A chi-square test showed a strong association between age group and content format preference, χ^2 ^(2, N = 507) = 112.57, p < 0.001, Cramer's V = 0.33. Younger participants (18-35 years) showed an overwhelming preference for short-form video content (selected by 78.2% of this cohort), with optimal engagement occurring with videos under three minutes in length. Older adults (55+ years) demonstrated a greater affinity for detailed surgeon testimonials and narrative case studies (63.4% preference). Geographic location emerged as a crucial factor, with a significant chi-square result showing rural respondents emphasised the need for offline-accessible materials more than urban residents, χ^2 ^(1, N = 507) = 205.18, p < 0.001, Cramer's V = 0.64 (91.3% vs. 18%). Educational attainment similarly influenced engagement patterns, as college-educated participants were 30% more likely to utilize interactive Question and Answer features than those with only secondary education, χ^2^ (1, N = 507) = 45.92, p < 0.001, Cramer's V = 0.30.

Surgical expert perspectives on knowledge gaps

In-depth interviews with nine robotic surgeons revealed three primary patient misconceptions about RAS. The most common misconception, reported by n = 8 (89%) of surgeons, was the belief that surgical robots operate autonomously.

Additionally, n = 6 (67%) of surgeons noted patient anxiety about potential mechanical failures, while n = 5 (56%) encountered skepticism regarding the advantages of RAS over traditional surgical methods. The interviews also highlighted significant gaps in surgical training, with early-career surgeons averaging only 12.4 console cases during their residency.

Digital education platform development

Based on these findings, we developed a comprehensive, three-tiered digital education framework. As shown in Table 3, the first tier, the Education Layer, was designed to address a majority of frequently asked questions with interactive 3D anatomical visualizations.

**Table 3:** Platform Component Validation Metrics S-CVI: Scale-Level Content Validity Index

Component	Reliability (Cronbach's α)	Content Validity S-CVI	Knowledge Improvement (%)
Education Layer	0.82	0.89	38
Interaction Layer	0.79	0.92	29
Decision Layer	0.85	0.93	42

The second, the Interaction Layer, offers patients the ability to connect with verified surgeons by accessing their profiles and provides a system for teleconsultation scheduling. Finally, the Decision Layer empowers patients to make informed choices with dynamic cost-comparison tools and provides clear visualizations of outcome data.

Platform efficacy and outcomes

Rigorous pilot testing of the digital education platform demonstrated significant improvements across all measured knowledge domains. Key results from the pilot efficacy test are summarized in Table 4.

**Table 4:** Efficacy of the Digital Education Platform (Pilot Test, N = 50) RAS: robotic-assisted surgery

Knowledge Domain	Pre-test Score, % correct (n)	Post-test Score, % correct (n)	Absolute Improvement
Overall Understanding	34.0 (n/a)	77.0 (n/a)	+43 percentage points
Surgeon's Role and Control	41.0 (21)	82.0 (41)	+41 percentage points
RAS Safety and Advantages	28.0 (14)	72.0 (36)	+44 percentage points

The platform's adaptive delivery system successfully accommodated diverse user needs, from tech-savvy urban millennials preferring on-demand micro learning content to older rural patients benefiting from more structured, narrative-driven educational pathways. These results demonstrate that carefully designed digital education can effectively bridge critical knowledge gaps in surgical innovation while addressing the varied needs of different patient populations across the healthcare spectrum.

## Discussion

A central finding of our study is the profound disparity in India between high public awareness of RAS (89.2%) and low functional understanding of its principles (17.3%). This gap is not merely a knowledge deficit but is actively populated by specific, persistent misconceptions. Our qualitative data reveal that the belief in fully autonomous robotic operation is the most common patient myth, reported by 89% of the surgeons we interviewed. This finding extends the global narrative, which notes similar fears of autonomy and technological failure in settings like the UK and Singapore [13,14], by quantifying the precise prevalence of this misconception within the Indian clinical encounter. Crucially, our survey further reveals that this understanding gap is significantly wider among rural and non-college-educated populations. This demographic patterning underscores that the barrier to informed consent is not just informational but is inextricably linked to broader issues of health literacy and equitable access to advanced medical knowledge in the Indian socio-economic context [15]. Therefore, our work demonstrates that effective patient education must be both myth-busting and deliberately tailored to bridge these structural divides.

The disparities we observed in RAS understanding across different demographic groups, specifically higher knowledge levels among urban, college-educated participants, further underscore the importance of tailored educational interventions. This finding is particularly pertinent when contrasted with the work of English et al. in 2024 [15], who defined health literacy as the ability to "obtain, engage, understand, and act upon health information." Their research highlights that low health literacy is a pervasive issue, with disproportionately higher rates among older and rural patients. Importantly, they link low health literacy to tangible negative outcomes, such as poor adherence to preoperative and discharge instructions and longer lengths-of-stay, suggesting a direct impact on surgical outcomes. Our study directly addresses this challenge by designing a digital platform that serves as a meaningful intervention at the patient level. By providing adaptive content delivery, including features like visual aids and offline access for rural areas, our approach aims to circumvent the geographic and educational barriers that contribute to low health literacy. This not only facilitates a better understanding of RAS but also promotes health equity by empowering diverse patient populations to more effectively engage with and act upon critical health information.

Beyond patient perceptions, our study provides original, quantitative insight into a critical systemic barrier: the stark deficit in structured RAS training. The early-career surgeons we interviewed reported an average of only 12.4 console cases, highlighting a training pipeline ill-prepared for technological adoption. This empirical data from India resonates with global analyses emphasizing the need for structured pathways to achieve proficiency [16]. However, our qualitative findings add a crucial dimension: surgeons explicitly linked this training gap to patient anxieties, noting that their own limited experience sometimes hindered their ability to convincingly advocate for RAS. Thus, our research uniquely connects the challenge of surgical training directly to the challenge of patient acceptance. The integration of verified surgeon profiles, including procedural volume, into our digital platform is a direct intervention designed to address this dual need by fostering transparency and trust based on demonstrated experience.

The successful pilot testing of our three-tiered digital education platform, validated by a significant increase in patient understanding, serves as the capstone of this research. The platform’s efficacy in addressing patient misconceptions and improving comprehension is a direct result of its evidence-based design. Our results are consistent with a qualitative evaluation by Nayak et al. in 2024 [17], which explored the acceptance of a robotic surgery education tool among the Indian public. They found that engaging, informative, and visually rich tools were crucial for building patient trust and promoting a positive attitude toward robotic surgery. The significant knowledge improvement observed in our pilot, particularly the 100% increase in understanding of surgeon control, confirms the effectiveness of our design philosophy. By leveraging interactive 3D anatomical visualizations, we directly countered the “autonomous robot” myth, a key finding identified by both our study and the broader literature. The platform’s ability to reduce cost-related misconceptions also addresses a practical barrier to adoption, consistent with research that identifies financial concerns as a major determinant in patient decision-making.

Finally, this research provides a comprehensive and locally grounded analysis of the barriers to RAS adoption, drawing on both quantitative data and the rich qualitative experiences of surgical professionals. By meticulously comparing and contrasting our findings with the provided literature, we have established that the challenges in India, from public misconceptions to training deficits, are part of a larger global narrative but are uniquely impacted by local socioeconomic factors. The development and pilot-testing of our digital platform offers a validated, scalable, and equitable solution to these challenges, proving that a well-designed educational strategy can be a powerful catalyst for informed decision-making and the successful, ethical integration of advanced surgical technologies.

Future research points

While this study provides a foundational understanding of the barriers to RAS adoption, several critical areas warrant further investigation. The next logical step is to conduct a large-scale randomized controlled trial to evaluate the long-term efficacy of the digital education platform, assessing not only sustained knowledge improvement but also its impact on patient-reported outcomes and RAS acceptance rates. Simultaneously, research should focus on developing and validating a standardized, competency-based training curriculum for RAS, using a multi-institutional approach to compare the proficiency and patient outcomes of surgeons trained with this new model versus traditional methods. A dedicated study is also needed to evaluate the real-world utility of the platform’s decision layer, specifically how cost and outcome comparison tools influence patient decision-making and whether they inadvertently introduce new ethical challenges. Finally, future research should expand its scope to include the perspectives of hospital administrators and policymakers, using qualitative methods to understand the institutional and financial barriers to RAS adoption, thereby ensuring that future solutions are not only clinically sound but also economically and logistically viable. Future research must empirically test the causal pathways suggested by this study. Specifically, longitudinal or interventional trials are needed to measure whether improving RAS knowledge via tools like our digital platform directly translates into increased acceptance rates or more shared decision-making, while controlling for the powerful confounding effects of cost, access, and institutional factors.

Study limitations

This study, while providing valuable insights, is subject to several limitations. The quantitative phase, relying on a convenience-based online survey, may not fully represent India’s diverse population, particularly individuals with limited digital access, potentially introducing a selection bias. The small sample size of nine surgeons in the qualitative component, while providing rich insights, limits the generalizability of their perspectives on training deficiencies and patient misconceptions to the entire surgical community. Furthermore, our pilot evaluation of the digital platform, while demonstrating a significant increase in immediate knowledge, was a short-term assessment that did not measure long-term information retention or the platform's sustained impact on patient decision-making and actual surgical outcomes. Finally, the study's scope was limited to patient and surgeon perspectives and did not explore the critical institutional, economic, and policy-related barriers, such as the high cost of robotic platforms and insurance coverage, that are also major determinants of RAS adoption. Furthermore, multiple statistical comparisons were made across demographic subgroups without correction, which increases the risk of type I error.

## Conclusions

The present research provides a comprehensive and locally grounded analysis of the barriers to RAS adoption, drawing on both quantitative data and the rich qualitative experiences of surgical professionals. By meticulously comparing and contrasting our findings with the provided literature, we have established that the challenges in India, from public misconceptions to training deficits, are part of a larger global narrative but are uniquely impacted by local socioeconomic factors. The development and pilot-testing of our digital platform offers a validated, scalable, and equitable solution to these challenges, proving that a well-designed educational strategy can be a powerful catalyst for informed decision-making and the successful, ethical integration of advanced surgical technologies.

## Appendices

Appendix A

National Survey on Robotic-Assisted Surgery (RAS) Awareness and Perceptions

Instrument description: This 35-item survey was developed to assess public awareness, knowledge, and perceptions of Robotic-Assisted Surgery (RAS) in India. It is structured into four primary domains. The instrument demonstrated acceptable content validity (S-CVI = 0.90) and internal consistency for the knowledge domain (Cronbach’s α = 0.78 in pilot testing).

Survey Items:

Domain 1: Demographic Information

Age: ( ) 18-22 ( ) 23-42 ( ) 43+

Gender: ( ) Male ( ) Female ( ) Non-binary / Prefer not to say

Geographic Location of Residence: ( ) Metropolitan City ( ) Tier-2 or Tier-3 City ( ) Semi-urban area ( ) Rural area

Highest Educational Qualification: ( ) Secondary school or below ( ) Undergraduate degree ( ) Postgraduate degree or above

Primary Occupation: ( ) Salaried professional ( ) Student ( ) Self-employed ( ) Homemaker ( ) Other (please specify): _______

Domain 2: General Awareness and Information Sources

6. Before today, were you aware of the existence of robotic-assisted surgery (RAS)?

( ) Yes ( ) No

7. What is your primary source of information about advanced medical technologies like RAS? (Select one)

( ) Television/Newspapers ( ) Social Media ( ) Healthcare Professionals ( ) Family/Friends ( ) Online Health Portals

8. How would you rate your general understanding of what robotic-assisted surgery entails?

( ) Very Poor ( ) Poor ( ) Fair ( ) Good ( ) Very Good

Domain 3: Knowledge of RAS Principles and Misconceptions

Instructions: Please indicate whether you believe the following statements are True or False.

9. In robotic-assisted surgery, the surgeon controls the robotic arms in real-time from a console in the operating room.

( ) True ( ) False ( ) Don't Know

10. The robotic system can operate on its own, making independent decisions during surgery.

( ) True ( ) False ( ) Don't Know

11. RAS procedures are typically "keyhole" or minimally invasive surgeries.

( ) True ( ) False ( ) Don't Know

12. The main advantage of RAS is that it completely replaces the need for a skilled surgeon.

( ) True ( ) False ( ) Don't Know

13. Robotic systems provide the surgeon with a high-definition, 3D view of the surgical area.

( ) True ( ) False ( ) Don't Know

14. RAS is available for a wide range of surgical specialties, including urology, gynecology, and general surgery.

( ) True ( ) False ( ) Don't Know

Domain 4: Educational Preferences and Decision-Making Factors

15. If you needed surgery and a robotic-assisted option was available, how likely would you be to consider it?

( ) Very Unlikely ( ) Unlikely ( ) Neutral ( ) Likely ( ) Very Likely

16. What factors would most influence your decision? (Select top two)

( ) Surgeon's recommendation ( ) Perceived accuracy/safety ( ) Cost ( ) Recovery time ( ) Fear of new technology

17. If seeking to learn more about RAS, what format would you prefer? (Select top two)

( ) Short animated videos (<3 mins) ( ) Detailed articles with images ( ) Patient testimonials/interviews ( ) Interactive 3D models ( ) Surgeon-led Q&A sessions

18. How important is it for a digital health education platform to be accessible offline (e.g., downloadable content)?

( ) Not Important ( ) Slightly Important ( ) Moderately Important ( ) Very Important ( ) Extremely Important

Appendix B

Knowledge Assessment for Digital Education Platform Pilot Test

Instrument description: This 10-item assessment was used in a pre-test/post-test design to evaluate the immediate efficacy of the digital education platform. It tests comprehension of key concepts directly addressed in the platform's content. The same instrument was administered before and after platform engagement.

Knowledge Assessment Items:

Instructions: Please choose the best answer for each question.

Who directly controls the robotic arms during a robotic-assisted surgery?

a) A pre-programmed computer

b) The surgeon from a console in the operating room

c) An assistant at the patient's bedside

d) The robot operates autonomously

What is a typical visual advantage for the surgeon using a robotic system?

a) A standard 2D television screen

b) A high-definition, magnified 3D view

c) No visual difference from traditional surgery

d) Direct line-of-sight only

How are robotic-assisted surgeries typically performed?

a) Through large, open incisions

b) Using "keyhole" or minimally invasive techniques

c) Without any incisions (non-invasive)

d) The approach is identical to traditional surgery

What happens if there is a technical failure (e.g., power loss) during a robotic procedure?

a) The surgery must be abandoned immediately

b) The surgeon can immediately convert to a traditional laparoscopic or open approach

c) The robot has a backup system to complete the surgery

d) The situation is always fatal

True or False: The primary goal of robotic surgery is to replace the surgeon.

a) True

b) False

Which of the following is a potential benefit of RAS over traditional methods?

a) Generally shorter operative times for all procedures

b) Consistently lower overall cost for the patient

c) Enhanced precision and dexterity for the surgeon

d) Elimination of all surgical risks

True or False: Robotic systems are used in only one or two specialized fields of surgery.

a) True

b) False

What role does the surgical team (other than the console surgeon) play during RAS?

a) They are not needed in the operating room.

b) They assist with patient-side tasks like instrument changes.

c) They program the robot before surgery and leave.

d) They take over if the surgeon gets tired.

True or False: Patient recovery time is usually longer after robotic surgery compared to open surgery.

a) True

b) False

For a patient, a key consideration when evaluating RAS should be:

a) The brand and model of the robot used.

b) The specific training, skill, and experience of the surgical team.

c) That it is always the best option for every type of surgery.

d) That it guarantees a perfect outcome.

Scoring Key:

1: b, 2: b, 3: b, 4: b, 5: b, 6: c, 7: b, 8: b, 9: b, 10: b.

One point is awarded for each correct answer. The total score is calculated as a percentage.

Appendix C

Platform Component Validation Metrics

This appendix provides the detailed reliability and validity metrics for the components of the three-tiered digital education platform, as derived from the expert validation and pilot testing phases. These data support the efficacy findings reported in the main manuscript.

**Table 5:** Validation Metrics for Digital Platform Components Scale-level Content Validity Index (S-CVI): Derived from expert review (five robotic surgeons, two patient education specialists). Represents the proportion of items rated as "highly relevant" or "relevant" (score of 4 or 5 on a 5-point Likert scale) for each platform layer. Cronbach’s α: Measured the internal consistency of user satisfaction survey items related to the perceived usefulness and usability of each specific layer during the pilot test (N = 50). Knowledge Improvement: The percentage point increase in correct responses in the pilot test's 10-item assessment for content domains covered by each respective layer (pre-test vs. post-test).

Platform Component (Layer)	Content Validity (S-CVI)	Internal Consistency of User Satisfaction Items (Cronbach’s α)	Measured Knowledge Improvement in Pilot Test (%)
Education Layer	0.89	0.82	+38
Interaction Layer	0.92	0.79	+29
Decision Layer	0.93	0.85	+42
